# Supplementary material for: TemStaPro: protein thermostability prediction using sequence representations from protein language models
Source: Bioinformatics. 2024 Mar 20;40(4):btae157. doi: 10.1093/bioinformatics/btae157 (PMC11001493; doi:10.1093/bioinformatics/btae157)
Supplement: btae157_Supplementary_Data [file btae157_supplementary_data.pdf]

Supplementary information for

TemStaPro: protein thermostability prediction using sequence representations from protein language models

Ieva Pudžiuvelytė [1,3], Kliment Olechnovič [1], Egle Godliauskaite [2], Kristupas Sermokas [2], Tomas Urbaitis [2], Giedrius Gasiunas [1,2], Darius Kazlauskas [1,*]

[1]Institute of Biotechnology, Life Sciences Center, Vilnius University, Saulėtekis av. 7, Vilnius, LT-10257, Lithuania
[2]CasZyme, Saulėtekis av. 7c, Vilnius, LT-10257, Lithuania
[3]Institute of Computer Science, Faculty of Mathematics and Informatics, Vilnius University, Didlaukio st. 47, Vilnius, LT-08303, Lithuania

[*]To whom correspondence should be addressed. Email: darius.kazlauskas@bti.vu.lt

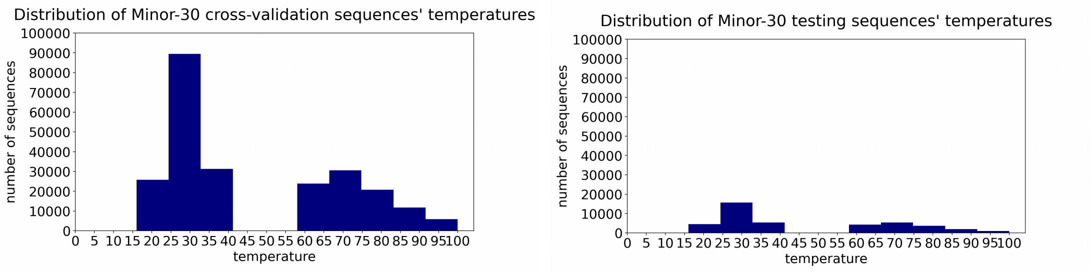

Figure S1. Protein sequences distribution regarding to organism's growth temperature in *TemStaPro-Minor-30* cross-validation and testing data subsets.

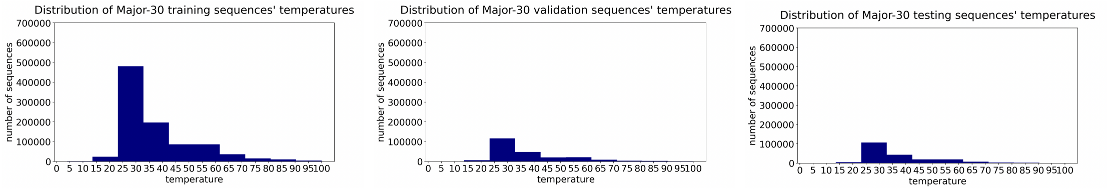

Figure S2. Protein sequences distribution regarding to organism's growth temperature in *TemStaPro-Major-30* data subsets.

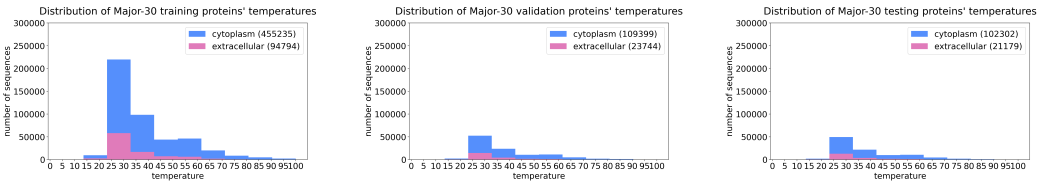

Figure S3. Distributions of protein sequences regarding to organism's growth temperature in *TemStaPro-Major-30* data subsets, plotted as overlapping histograms for protein sequences predicted to belong to either *cytoplasm* or *extracellular* class, number of sequences of each group is indicated in the brackets.

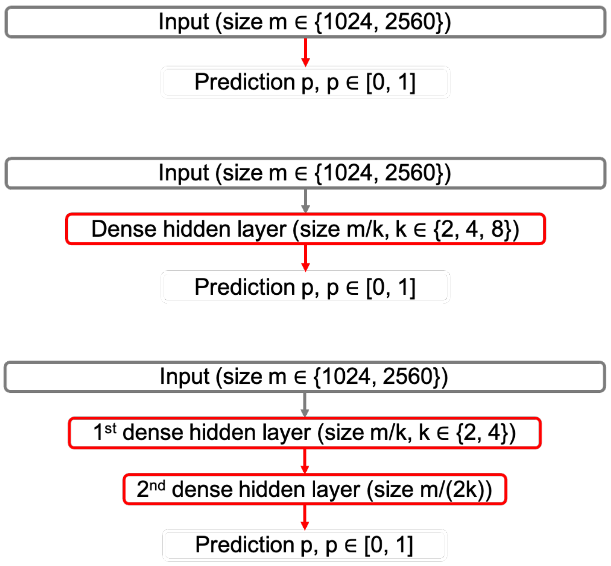

Figure S4. Schemes of architectures: single-layer perceptron (upper), a feed-forward neural network model with 1 hidden layer (middle), and a feed-forward neural network model with 2 hidden layers (lower).

Table S1. Models that were tested with ESM-2 and ProtT5-XL embeddings as input.

Model	Number of hidden layers	Size of hidden layers
C2H2_h1280-640	2	1280, 640
C2H2_h640-320	2	640, 320
C2H1_h1280	1	1280
C2H1_h640	1	640
C2H1_h320	1	320
SLP_ESM-2	0	-

Model	Number of hidden layers	Size of hidden layers
C2H2_h512-256	2	512, 256
C2H2_h256-128	2	256, 128
C2H1_h512	1	512
C2H1_h256	1	256
C2H1_h128	1	128
SLP_ProtT5-XL	0	-

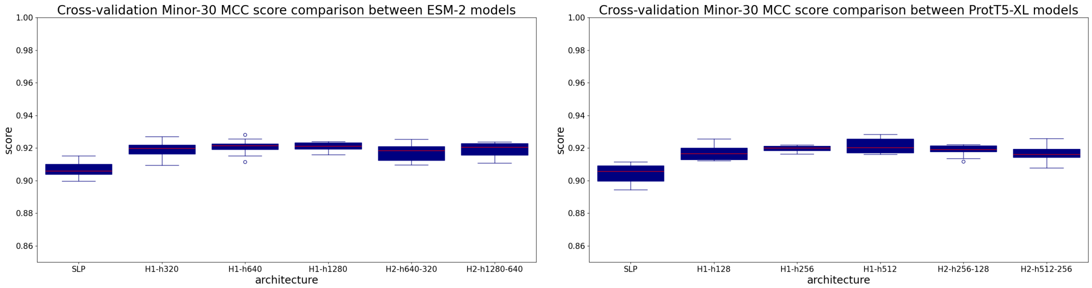

Figure S5. Cross-validation MCC scores of different architecture models', which were trained using ESM-2 or ProtT5-XL embeddings of *TemStaPro-Minor-30* set (weight decay equals 0).

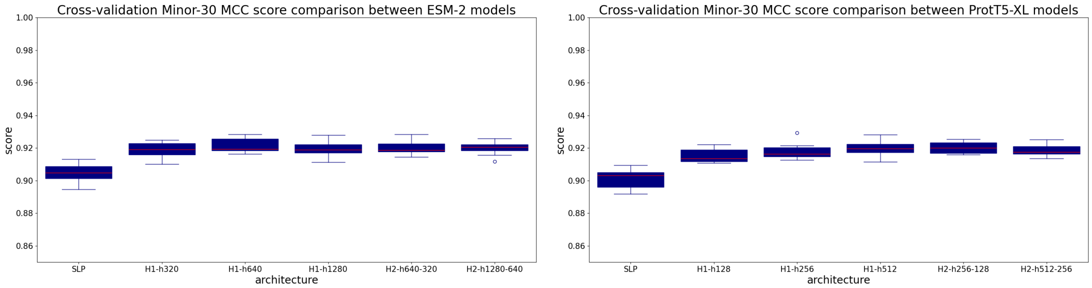

Figure S6. Cross-validation MCC scores of different architecture models', which were trained using ESM-2 or ProtT5-XL embeddings of *TemStaPro-Minor-30* set (weight decay 10^{-4}).

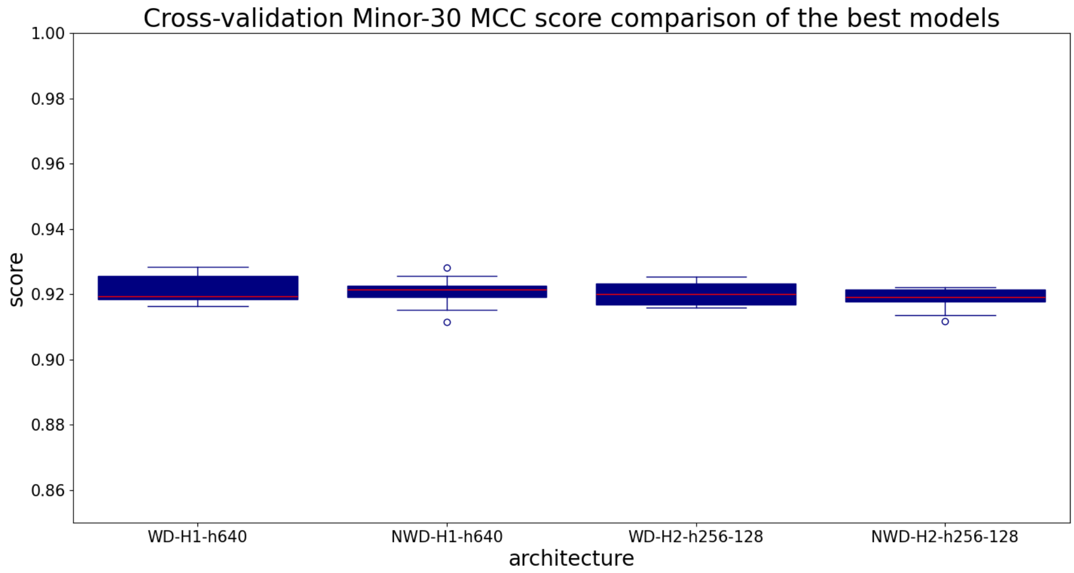

Figure S7. Comparison of weight decay effect on the best architectures' (after cross-validation) MCC scores.

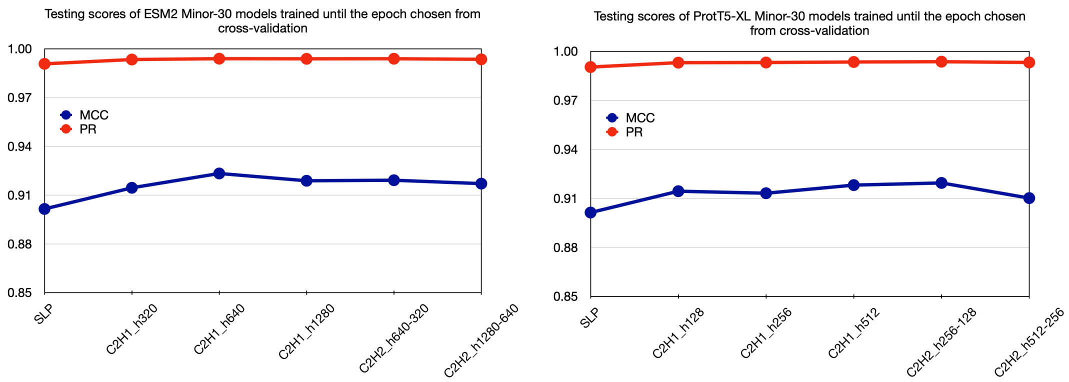

Figure S8. Comparison of different architecture models', which were trained using ESM-2 or ProtT5-XL embeddings, MCC and PR AUC scores for *TemStaPro-Minor-30* testing set.

Table S2. Model ensembles' scores after testing with the *TemStaPro-Major-30* dataset.

Model	MCC	Accuracy	Precision	Specificity	Recall	ROC AUC	F1	PR AUC
TemStaPro-t80	0.515	0.947	0.295	0.947	0.956	0.990	0.450	0.768
TemStaPro-t75	0.543	0.940	0.335	0.939	0.947	0.987	0.495	0.780
TemStaPro-t70	0.584	0.926	0.398	0.925	0.938	0.982	0.559	0.808
TemStaPro-t65	0.610	0.919	0.449	0.919	0.922	0.978	0.604	0.820
TemStaPro-t60	0.601	0.888	0.468	0.886	0.905	0.964	0.617	0.804
TemStaPro-t55	0.613	0.860	0.529	0.855	0.888	0.948	0.663	0.814
TemStaPro-t50	0.638	0.856	0.605	0.854	0.865	0.939	0.712	0.838
TemStaPro-t45	0.625	0.843	0.651	0.852	0.818	0.918	0.725	0.836
TemStaPro-t40	0.622	0.833	0.677	0.842	0.811	0.910	0.738	0.845

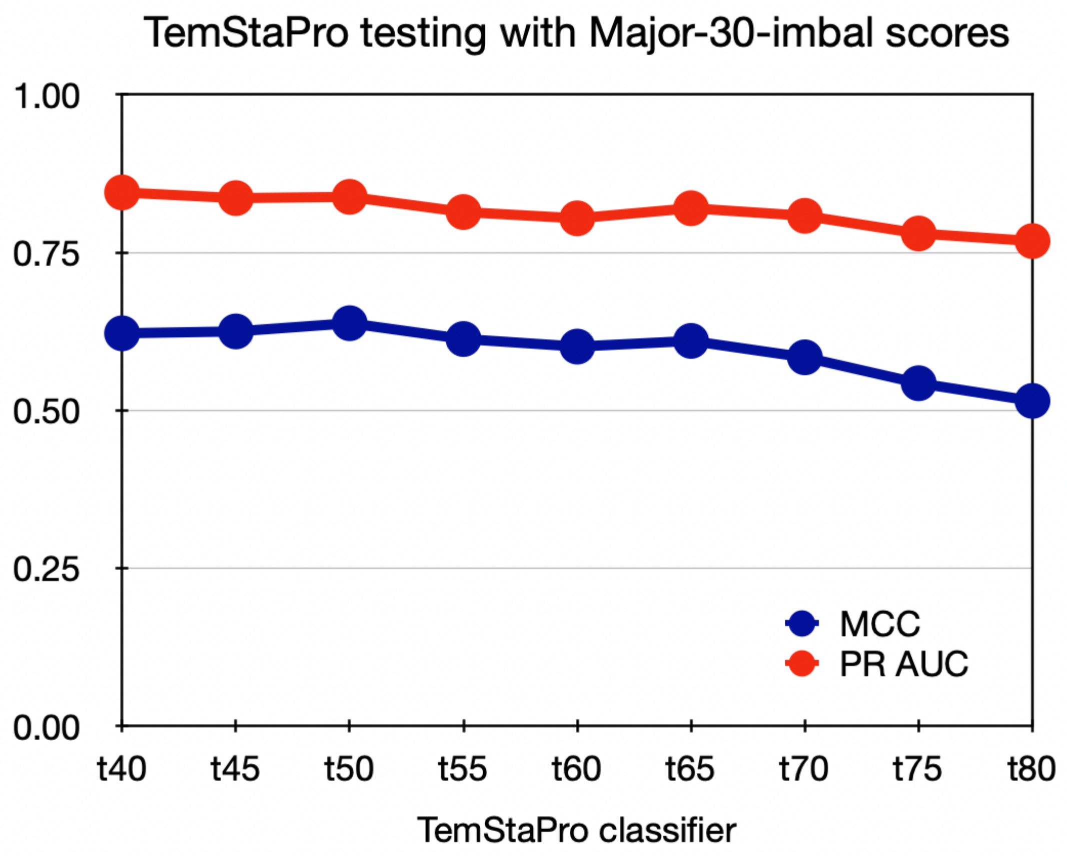

Figure S9. Model ensembles' MCC scores after testing with the *TemStaPro-Major-30* dataset.

Table S3. Comparison of BertThermo, ProLaTherm, and TemStaPro classifiers' scores using *TemStaPro-Major-30-sample2k* datasets.

Model	Dataset	MCC	Accuracy	Precision	Specificity	Recall	ROC AUC	F1	PR AUC
TemStaPro-t80	bal80	0.903	0.952	0.947	0.946	0.957	0.992	0.952	0.991
TemStaPro-t75	bal75	0.892	0.946	0.943	0.943	0.949	0.988	0.946	0.986
TemStaPro-t70	bal70	0.857	0.928	0.928	0.928	0.929	0.982	0.929	0.982
TemStaPro-t65	bal65	0.811	0.905	0.906	0.906	0.905	0.974	0.905	0.974
TemStaPro-t60	bal60	0.805	0.902	0.897	0.896	0.909	0.969	0.903	0.969
TemStaPro-t55	bal55	0.732	0.866	0.857	0.854	0.878	0.948	0.868	0.945
TemStaPro-t50	bal50	0.712	0.856	0.836	0.826	0.885	0.935	0.860	0.936
TemStaPro-t45	bal45	0.663	0.832	0.839	0.842	0.821	0.919	0.830	0.927
TemStaPro-t40	bal40	0.633	0.817	0.818	0.819	0.814	0.908	0.816	0.917
BertThermo	bal80	0.700	0.848	0.810	0.787	0.908	0.847	0.856	0.882
	bal75	0.690	0.840	0.788	0.751	0.928	0.839	0.853	0.876
	bal70	0.622	0.809	0.782	0.761	0.858	0.810	0.818	0.856
	bal65	0.624	0.811	0.792	0.778	0.845	0.811	0.818	0.857
	bal60	0.590	0.795	0.792	0.790	0.800	0.795	0.796	0.846
	bal55	0.521	0.756	0.810	0.843	0.670	0.756	0.733	0.823
	bal50	0.446	0.718	0.775	0.821	0.615	0.718	0.686	0.791
	bal45	0.442	0.710	0.806	0.867	0.553	0.710	0.656	0.791
	bal40	0.385	0.683	0.767	0.840	0.526	0.683	0.624	0.765
ProLaTherm	bal80	0.830	0.914	0.894	0.888	0.941	0.914	0.917	0.932
	bal75	0.797	0.898	0.891	0.889	0.908	0.899	0.899	0.923
	bal70	0.756	0.877	0.892	0.896	0.859	0.878	0.875	0.911
	bal65	0.732	0.866	0.886	0.892	0.839	0.865	0.862	0.903
	bal60	0.704	0.847	0.919	0.933	0.761	0.847	0.833	0.900
	bal55	0.580	0.772	0.916	0.945	0.599	0.772	0.724	0.858
	bal50	0.510	0.730	0.906	0.947	0.512	0.730	0.654	0.831
	bal45	0.482	0.705	0.932	0.968	0.442	0.705	0.600	0.827
	bal40	0.434	0.679	0.911	0.961	0.398	0.680	0.554	0.805

protein_id	position	sequence	length	t40_binary	t40_raw	t45_binary	t45_raw	t50_binary	t50_raw	t55_binary	t55_raw	t60_binary	t60_raw	t65_binary	t65_raw	left_hand_label	right_hand_label	clash
YmeCas12a	-	MSKVNNGF...FVLRNLS	1362	0	2.324E-01	0	2.806E-01	0	7.563E-02	0	5.726E-02	0	1.539E-02	0	1.216E-02	<40	<40	-
SauCas9	-	MKRNYIL...PQIIKKG	1053	0	4.03E-01	1	5.371E-01	0	3.865E-01	0	2.999E-01	0	8.613E-02	0	1.718E-02	<40	[45-50)	*
SpyCas9	-	MDKKYSI...LSQLGGD	1368	0	6.128E-02	0	4.68E-02	0	1.984E-02	0	2.198E-02	0	9.72E-03	0	1.842E-03	<40	<40	-
CaldoCas9	-	MRYKIGL...PLQSTRD	1087	1	6.278E-01	1	8.361E-01	1	7.301E-01	1	6.187E-01	0	4.024E-01	0	2.872E-02	[55-60)	[55-60)	-

Figure S10. An example tab-separated table that is the output of the (default) global prediction mode of TemStaPro program. The main output of the method is a TSV table with 8 columns: 'protein_id' - a header taken from the FASTA file of the input protein; 'sequence' - an amino acid sequence of the protein; 'length' - a length of the protein's amino acid sequence; 't??_binary' - a binary prediction label for a given temperature threshold (one of the six thresholds is written in the place of question marks) - the label is assigned by rounding the raw prediction (see the next point) at this temperature threshold; 't??_raw' - a raw prediction value for a given temperature threshold (real numbers from the interval [0, 1]); 'left_hand_label' - a label of the highest temperature range, at which the protein was predicted to still be thermostable (possible labels of temperature ranges are: '<40', '[40-45)', '[45-50)', '[50-55)', '[55-60), '[60-65)', '$\geq$65'); 'right_hand_label' - a label that is interpreted as 'left_hand_label', yet the label is assigned by reading the outputs starting from the right (possible values of the label coincide with the 'left_hand_label'); 'clash' - a Boolean identifier, whether a contradiction between the models' predictions was observed - the expected output is a decreasing sequence of binary predictions if the outputs are read from left to right in the increasing order of the temperature thresholds (expected output is labelled as '-' and other cases are assigned '*').

protein_id	position	sequence	length	t40_binary	t40_raw	t45_binary	t45_raw	t50_binary	t50_raw	t55_binary	t55_raw	t60_binary	t60_raw	t65_binary	t65_raw	left_hand_label	right_hand_label	clash
YmeCas12a	-	MSKVNNG...FVLRNLS	1362	0	3.663E-01	0	2.853E-01	0	1.332E-01	0	6.786E-02	0	1.174E-02	0	1.024E-02	<40	<40	-
YmeCas12a	1	M	1	0	9.439E-02	0	3.06E-01	0	7.553E-02	0	2.346E-03	0	1.262E-02	0	4.2E-03	<40	<40	-
YmeCas12a	2	S	1	0	5.779E-05	0	9.265E-06	0	1.332E-07	0	7.257E-07	0	8.085E-04	0	8.957E-02	<40	<40	-
YmeCas12a	3	K	1	1	6.523E-01	1	6.74E-01	0	2.265E-02	0	1.152E-01	0	6.501E-02	0	9.952E-02	[45-50)	[45-50)	-
YmeCas12a	4	V	1	1	9.991E-01	1	9.998E-01	0	4.998E-01	0	6.988E-05	1	7.067E-01	0	3.767E-01	[45-50)	[60-65)	*
YmeCas12a	5	N	1	0	2.412E-01	0	4.262E-01	0	1.075E-03	0	5.545E-05	0	6.789E-08	0	1.216E-05	<40	<40	-
YmeCas12a	6	N	1	1	9.898E-01	1	9.989E-01	0	3.282E-01	1	5.728E-01	0	4.837E-01	0	3.104E-01	[45-50)	[55-60)	*
YmeCas12a	7	G	1	1	8.477E-01	1	9.896E-01	0	6.323E-02	0	2.215E-02	0	9.535E-06	0	4.163E-06	[45-50)	[45-50)	-
YmeCas12a	1356	F	1	1	9.98E-01	1	9.991E-01	1	9.971E-01	1	9.946E-01	1	9.872E-01	1	9.906E-01	65<=	65<=	-
YmeCas12a	1357	V	1	1	9.996E-01	1	1E+00	1	9.993E-01	1	9.982E-01	1	9.917E-01	1	9.451E-01	65<=	65<=	-
YmeCas12a	1358	L	1	0	2.587E-03	0	2.595E-01	0	4.147E-01	0	4.249E-01	1	8.449E-01	1	9.596E-01	<40	65<=	*
YmeCas12a	1359	R	1	1	7.469E-01	1	7.959E-01	1	9.699E-01	1	7.673E-01	1	8.938E-01	0	4.692E-02	[60-65)	[60-65)	-
YmeCas12a	1360	N	1	0	7.824E-03	0	3.935E-03	0	7.495E-03	0	8.975E-02	0	8.908E-03	0	3.43E-01	<40	<40	-
YmeCas12a	1361	L	1	0	3.454E-06	0	2.247E-06	0	9.429E-06	0	1.137E-05	0	1.454E-03	0	5.699E-03	<40	<40	-
YmeCas12a	1362	S	1	0	5.388E-04	0	1.277E-02	0	8.619E-03	0	1.917E-01	0	1.648E-03	0	5.585E-02	<40	<40	-

Figure S11. An example tab-separated table that is the output of per-residue prediction mode of TemStaPro program.

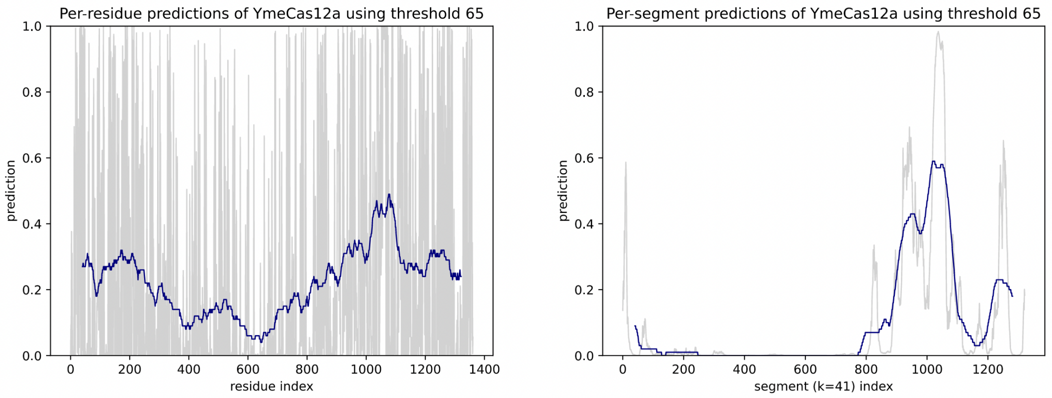

Figure S12. An example plot for the output of per-residue mode (left) and per-segment mode with default window size of 41 (right).

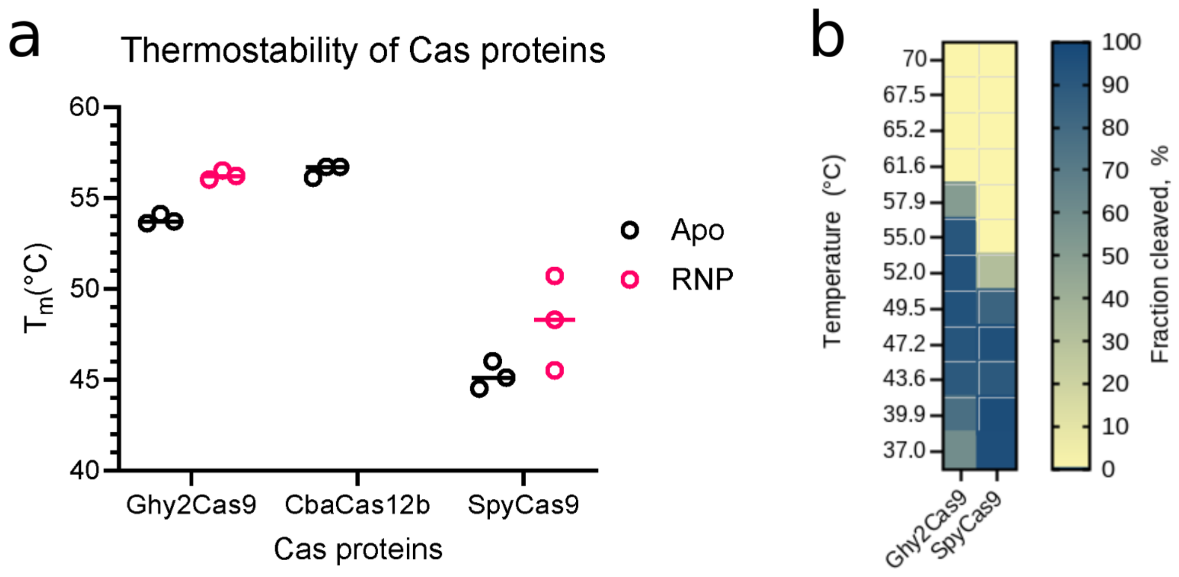

Figure S13. **(a)** Thermal stability of Cas proteins without guide RNA (apo) or loaded with sgRNA (Ghy2Cas9 and SpyCas9) (RNP). Protein unfolding was measured using nano differential scanning fluorimetry (nanoDSF) over a temperature range from 20 °C to 80 °C. Fluorescence was monitored as temperature increased at a rate of 1 °C per second. The inflection point of the fluorescent curve is interpreted as the unfolding point of the protein (T_m). Data points collected from replicate experiments are plotted as circles, the means are plotted as dashes. **(b)** The double-stranded DNA (dsDNA) cleavage activities of Ghy2Cas9 and SpyCas9 RNPs were measured using in vitro assays containing fluorophore-labeled dsDNA target substrates. Cleaved fragments were quantitated and are represented in a heatmap showing overall activity at temperatures ranging from 37 °C to 70 °C. The intensity of the blue colour indicates the fraction of substrate cleaved.

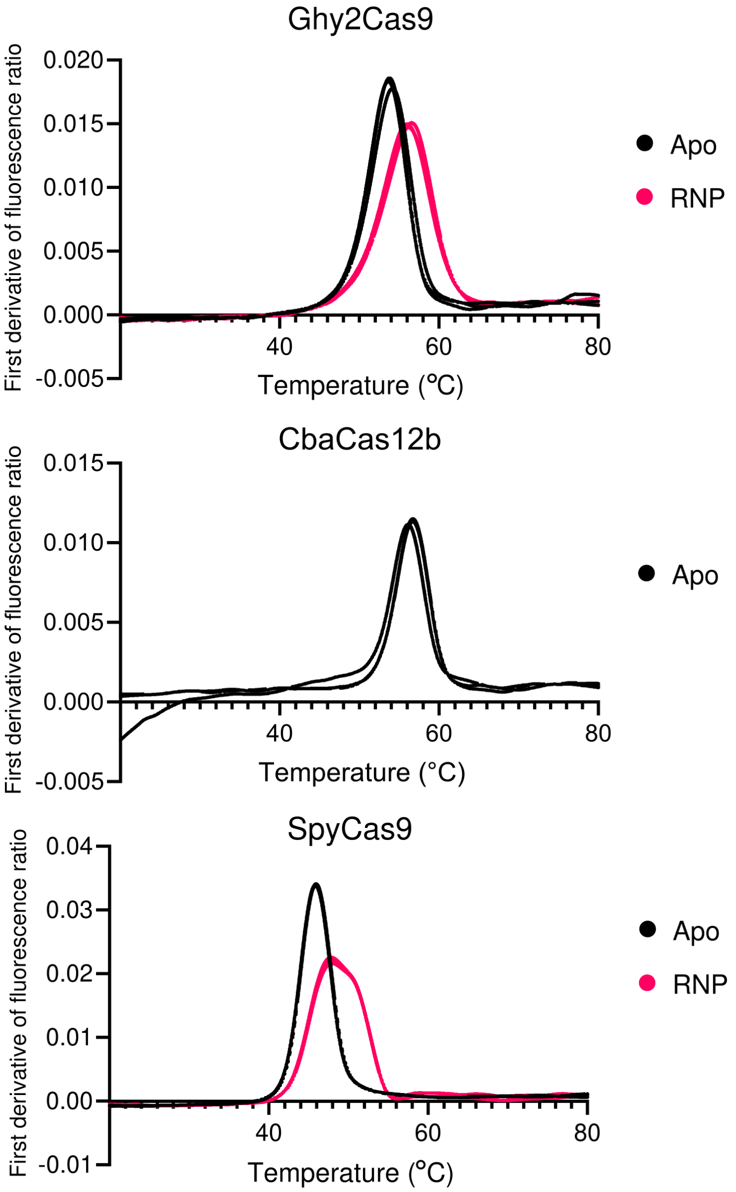

Figure S14. Raw traces of nanoDSF assays (*SupplementaryMeltingTemperatureSourceData.xlsx*). The first derivative of the fluorescence ratio was graphed using the integrated fluorescence data at 350 nm and 330 nm by the Prometheus instrument during experiments where the temperature was increased at the rate of 1 °C per second for Cas proteins without guide RNA (apo) or with guide RNA (RNP). The peaks in the first derivative graph are inflection points that correspond to the temperature at which half of the molecules have undergone transition to a new state, which is interpreted as the protein unfolding (denaturing or melting).

Table S4. Experimentally characterized and predicted temperatures of C2EP proteins. The full table is in *SupplementaryTableCharacterizedC2EPs.xlsx*.

Group	Name	Length	Max. active temp	Max. optimal active temperature	Melting point	Reference PubMedID	Prediction left-hand label	Prediction right-hand label	Prediction clash	Difference more than 10 °C[2]
Cas12a	YmeCas12a	1362	60	-	60	27989439	<40	<40	-	Yes
Cas12a	CmeCas12a	1288	55	-	55	27989439	<40	<40	-	Yes
Cas12a	RbCas12a	1247	42	-	45	36012553	<40	<40	-	No
Cas12b	AacCas12b	1129	55	-	-	27989439	[45-50)	[45-50)	-	No
Cas12b	BrCas12b	1090	63	-	-	29127284	[55-60)	[55-60)	-	No
Cas12b	AaCas12b	1129	65	59	-	30510770	[50-55)	[50-55)	-	No
Cas12b	AacC2c1	1129	60	-	-	27984729	[55-60)	[55-60)	-	No
Cas12b	CbaCas12b	1120	-	-	57	This study	[60-65)	[60-65)	-	No
Cas13a	TccCas13a	1225	70	-	-	35763567	[40-45)	[40-45)	-	Yes
Cas13x	mCas13	873	50	37	-	34546709	<40	<40	-	No
Cas9-C1	VpaCas9	1398	48	-	43	33139742	<40	<40	-	No
Cas9-C1	KhuCas9	1309	46	-	33	33139742	<40	<40	-	No
Cas9-C1	FmaCas9	1348	44	-	41	33139742	<40	[45-50)	*	-
Cas9-C1	EitCas9	1330	44	-	37	33139742	<40	<40	-	No
Cas9-C1	Sag1Cas9	1384	44	-	40	33139742	<40	<40	-	No
Cas9-C1	Sag2Cas9	1377	41	-	41	33139742	<40	<40	-	No
Cas9-C1	SdyCas9	1371	48	-	45	33139742	<40	<40	-	No
Cas9-C1	SmuCas9	1345	48	-	45	33139742	<40	<40	-	No
Cas9-C1	SpyCas9	1368	50	42	47	28146359	<40	<40	-	No
Cas9-C2	Cme3Cas9	1124	54	-	50	33139742	[50-55)	[50-55)	-	No
Cas9-C2	Ghh1Cas9	1094	37	-	41	33139742	<40	<40	-	No
Cas9-C2	CgaCas9	1403	37	-	36	33139742	<40	<40	-	No
Cas9-C2	CcalCas9	1430	44	-	41	33139742	<40	<40	-	No
Cas9-C2	Cme1Cas9	1399	41	-	47	33139742	<40	<40	-	No
Cas9-C2	OrhCas9	1535	44	-	43	33139742	<40	<40	-	No
Cas9-C2	WviCas9	1440	48	-	43	33139742	<40	<40	-	No
Cas9-C2	Ghy2Cas9	1358	58	55	57	This study	[55-60)	[55-60)	-	No
Cas9-C2	IgnaviCas9	1244	90	-	-	31659048	[55-60)	[55-60)	-	Yes
Cas9-C3	TmoCas9	1049	46	37	48	33139742	<40	<40	-	No
Cas9-C3	Ghy3Cas9[1]	972	46	-	-	33139742	[55-60)	[55-60)	-	No
Cas9-C3	CaldoCas9	1087	60	-	-	33953310	[55-60)	[55-60)	-	No
Cas9-C3	GeoCas9	1087	65	-	-	29127284	[55-60)	[55-60)	-	No
Cas9-C3	ThermoCas9	1082	60	-	-	29162801	[55-60)	[55-60)	-	No
Cas9-C4	Sth1ACas9	1122	57	-	50	33139742	<40	<40	-	No
Cas9-C4	SauCas9	1053	50	-	50	33139742	<40	[45-50)	*	-
Cas9-C4	MgaCas9	1269	46	44	47	33139742	<40	<40	-	No
Cas9-C4	SsaCas9	1127	50	-	41	33139742	<40	<40	-	No
Cas9-C4	SsiCas9	1122	48	-	44	33139742	<40	<40	-	No
Cas9-C4	SsuCas9	1122	44	-	42	33139742	<40	<40	-	No
Cas9-C5	AceCas9	1138	60	50	-	28277645	<40	<40	-	No
Cas9-C7	NsaCas9	1137	64	-	67	33139742	[60-65)	[60-65)	-	No

[1] - mislabeling in Fig. 4A of 33139742 instead of Ghy2, should be Ghy3;
[2] - prediction differs more than 10 °C from max active/optimal temperature and protein melting point.
